# Misconduct Policies in High-Impact Biomedical Journals

**DOI:** 10.1371/journal.pone.0051928

**Published:** 2012-12-19

**Authors:** Xavier Bosch, Cristina Hernández, Juan M. Pericas, Pamela Doti, Ana Marušić

**Affiliations:** 1 Department of Internal Medicine, Institut d’Investigacions Biomèdiques August Pi i Sunyer, Hospital Clínic, University of Barcelona, Barcelona, Spain; 2 Department of Research in Biomedicine and Health, University of Split School of Medicine, Split, Croatia; University of Chieti, Italy

## Abstract

**Background:**

It is not clear which research misconduct policies are adopted by biomedical journals. This study assessed the prevalence and content policies of the most influential biomedical journals on misconduct and procedures for handling and responding to allegations of misconduct.

**Methods:**

We conducted a cross-sectional study of misconduct policies of 399 high-impact biomedical journals in 27 biomedical categories of the Journal Citation Reports in December 2011. Journal websites were reviewed for information relevant to misconduct policies.

**Results:**

Of 399 journals, 140 (35.1%) provided explicit definitions of misconduct. Falsification was explicitly mentioned by 113 (28.3%) journals, fabrication by 104 (26.1%), plagiarism by 224 (56.1%), duplication by 242 (60.7%) and image manipulation by 154 (38.6%). Procedures for responding to misconduct were described in 179 (44.9%) websites, including retraction, (30.8%) and expression of concern (16.3%). Plagiarism-checking services were used by 112 (28.1%) journals. The prevalences of all types of misconduct policies were higher in journals that endorsed any policy from editors’ associations, Office of Research Integrity or professional societies compared to those that did not state adherence to these policy-producing bodies. Elsevier and Wiley-Blackwell had the most journals included (22.6% and 14.8%, respectively), with Wiley journals having greater a prevalence of misconduct definition and policies on falsification, fabrication and expression of concern and Elsevier of plagiarism-checking services.

**Conclusions:**

Only a third of top-ranking peer-reviewed journals had publicly-available definitions of misconduct and less than a half described procedures for handling allegations of misconduct. As endorsement of international policies from policy-producing bodies was positively associated with implementation of policies and procedures, journals and their publishers should standardize their policies globally in order to increase public trust in the integrity of the published record in biomedicine.

## Introduction

Journals are vital to research integrity, and, through peer review, an essential means of communication between scientists. However, they are also a potential source of scientific error if articles are tainted by research misconduct.

As the problem has been known for many years, journal editors might be expected to have misconduct policies in place today. If not, perhaps they do not regard misconduct as a serious problem. In addition, it may be difficult, time-consuming and legally-challenging to deal with misconduct in published articles [1].

The US Office of Research Integrity (ORI) defines research misconduct as “fabrication, falsification, or plagiarism in proposing, performing, or reviewing research, or in reporting research results”. Fabrication is defined as “making up data or results and recording or reporting them”; falsification is defined as “manipulating research materials, equipment, or processes, or changing or omitting data or results such that the research is not accurately represented in the research record”; and plagiarism is defined as “the appropriation of another person’s ideas, processes, results, or words without giving appropriate credit” [2].

Editorial associations, such as the Committee on Publication Ethics (COPE), International Committee of Medical Journal Editors (ICMJE) and World Association of Medical Editors have produced guidelines on the responsibility of journal editors when research misconduct is suspected or confirmed in published or submitted articles. Likewise, the 2012 Council of Science Editors’ ‘White Paper on Promoting Integrity in Scientific Journal Publications’ defined misconduct and suggested how journals should treat it [3]. Some publishers have also made their position clear, including Wiley-Blackwell, who issued the position statement ‘Best Practice Guidelines on Publication Ethics: A Publisher’s Perspective’ [4]. Public bodies, notably the ORI, have also formulated recommendations for journals to develop policies on misconduct [5]. In particular, the ORI first addressed this issue in January 2000 in its ‘Guidance Document for Editors’, whose objective was to guide journal editors and staff on the reporting of suspect manuscripts, the investigation of allegations of misconduct and, in general, ensure the integrity of research [5]. This document was the catalyst for the development of policies by professional associations.

As far as we are aware, the first time this topic was addressed and published was at the 1st Research Conference on Research Integrity, sponsored by the ORI in November 2000. The resulting report by Scheetz reviewed the subjects addressed by author instructions other than preparation of the manuscript, most notably research integrity [6]. The authors studied the instructions to authors of 41 journals that were requested to publish corrections or retractions due to research misconduct between 1992 and 1999. The study found that most issues received only minimal consideration in the authors instructions, with only around 14% of the content of instructions relating to concerns about research integrity (principally correction of the literature) and the rest concentrating overwhelmingly on the preparation of manuscripts.

Although biomedical journals have taken a leading position in formulating editorial policies [7], there is little evidence on which policies are in place in biomedical journals to deal with misconduct and available to the public and prospective authors.

Why is the lack of scientific misconduct policies an issue? In essence, the absence or lack of policies has perpetuated a *de novo* approach for many journals, which has contributed to publication delays and stress, and fueled many preventable legal battles.

Without policies, journals have to react *ad hoc* to allegations and perhaps enter into legal disputes. Clear and public policies are the best method of preventing complications after allegations are made. The current dearth of uniform use of such policies in journals is an issue for editors, publishers, authors, their institutions and all other stakeholders in research.

The aim of this study was to assess the prevalence and content policies of the most influential biomedical journals on misconduct and procedures for handling and responding to allegations of misconduct.

## Methods

### Study Design

We performed a cross-sectional study of misconduct policies of peer-reviewed biomedical journals.

### Sample Selection

We selected a broad sample of peer-reviewed journals, reflecting a wide range of biomedical research fields.

We included the 15 top-rated journals from 27 categories of the Journal Citation Reports (JCR) (all 10 and 14 journals for the categories “Cell and Tissue Engineering” and “Biology”, respectively). The total sample comprised 399 journals (Table S1). Journals were rated using the 2010 impact factor (IF) published by the Institute for Scientific Information JCR [8]. Journals belonging to more than one category were included in the category with the highest rank and another journal was included in the other categories.

We included English-language journals publishing research studies. Seventy-nine journals publishing review articles and no original research were excluded.

### Data Collection and Analysis

Instructions for authors and manuscript submission documents were collected, including any guidelines or instructions pertaining to editorial policies or manuscript submission and all available documents related to manuscript submission relevant to research misconduct. All information collected was publicly available on journal websites. Each journal website was reviewed to find information relevant to misconduct policies, which we defined as rules or statements about the definition of misconduct or procedures for responding to misconduct. The search strategy included, but was not limited to, the following search terms (modified from Steneck, Office of Research Integrity, USA [9]) misconduct, ethics, falsification, fabrication, fraud, plagiarism, duplication, overlapping publication, redundant publication, image manipulation and integrity. We also recorded whether journals had procedures for responding to misconduct including, but not limited to, publishing an expression of concern and/or retraction, and whether plagiarism-checking services were routinely used. We did not consider journal policies pertaining to simultaneous/dual submission to be misconduct policies. Policies on authorship, conflicts of interest and ghostwriting were not considered for this study.

Information for each journal was reviewed independently by two authors (CH, JP or PD) using a standard form (Appendix S1) in December 2011.

The following information was collected for each journal: 2010 IF, medical category, editorial office site, type of contents (basic research, clinical research or both) and publisher, including for-profit and non-for profit publishers and professional societies.

We also recorded whether misconduct policies were generated by 1) the journal itself, 2) editors’ associations, ORI or professional societies (e.g., American Diabetes Association), or 3) the journal publisher. For clarity, we use the term ‘policy-producing bodies’ to refer to editors’ associations, ORI and professional societies that have created misconduct guidelines. We limited our analysis to 7 major publishers, publishing 257 (64.6%) journals, while the rest were categorized as ‘other’ (Table 1).

**Table 1:** Characteristics of the journals included in the study.

Characteristics	Number of journals n = 399 (%)
Editorial office site	
○USA	220 (55.1)
○Europe	132 (33.1)
○Europe and USA	23 (5.8)
○Canada	10 (2.5)
○Other countries	14 (3.5)
Type of contents	
○Clinical	123 (30.8)
○Basic	162 (40.6)
○Clinical and basic	114 (28.6)
Publisher	
○Elsevier	90 (22.6)
○Wiley-Blackwell	59 (14.8)
○Springer	29 (7.3)
○Nature	28 (7.0)
○Lippincott WW	23 (5.8)
○Oxford Journals	21 (5.3)
○Mary Ann Liebert	7 (1.8)
○Other	142 (35.6)
Endorsement of policy-producing body guidelines	
○ICMJE	159 (39.8)
○COPE	157 (39.3)
○WAME	44 (11.0)
○ORI	32 (8.0)
○CSE	6 (1.5)
○Other bodies *	21 (5.3)
Source of misconduct policies	
○Journal	132 (33.1)
○Publisher	143 (35.8)
○Editors’ associations/ORI/professional societies	124 (31.1)
Misconduct policies	
○Mention of misconduct	279 (69.9)
○Definition of misconduct	140 (35.1)
○Falsification	113 (28.3)
○Fabrication	104 (26.1)
○Plagiarism	224 (56.1)
○Duplication	242 (60.7)
○Image manipulation	69 (17.3)
○Other forms of misconduct †	154 (38.6)
Plagiarism-checking service	112 (28.1)
Procedures for responding to	179 (44.9)
○Retraction	123 (30.8)
○Expression of concern	65 (16.3)
○Other procedures ‡	130 (32.6)

* Mainly professional societies such as British Cardiovascular Society, American Diabetes Association and The Endocrine Society.

† Examples include, among others, ‘suppression of undesirable results’, ‘multiple publication’ and ‘misinterpretation of results’.

‡ Examples include, among others, ‘publish complete errata’, ‘letter of explanation’ and ‘letter of reprimand’.

### Statistical Analysis

Categorical variables were described using frequencies and percentages, and the IF, which is a quantitative variable, using the mean and standard deviation, median and 25% and 75% percentiles. The Chi-square test or Fisher’s exact test were used to compare categorical variables, as appropriate. To analyze the relationship between the IF and study variables, the non-parametric Mann-Whitney U test was used for comparisons between groups. The level of statistical significance was established at 5% bilateral. The analysis was made using the PASW 18.0 statistical system (SPSS, Chicago, Illinois, USA).

## Results

Of the 399 journals analyzed, 162 (40.6%) published basic research, 123 (30.8%) clinical research and 114 (28.6%) both, and 132 (33.1%) journals were based in Europe and 220 (55.1%) in the USA (Table 1). The mean IF of the journals was 6.51 (standard deviation: 5.49).

Definitions or guidelines of policy-producing bodies were endorsed by 239 (59.9%) journals. Misconduct policies were generated by the journal itself in 132 (33.1%) cases and by the publisher in 143 (35.8%), whereas124 (31.1%) journals stated that their policies were adopted from policy-producing bodies.

The term ‘misconduct’ was mentioned in the web-documents of 279 (69.9%) journals and 140 (35.1%) journals provided explicit definitions of misconduct (Table 1); of these, 10 of 15 journals were from the Gastroenterology and Metabolism category, 9 of 15 from Endocrinology and Metabolism, 8 of 15 from Medicine, General and Internal and 8 of 15 from Biochemistry and Molecular Biology. In contrast, only 1 of 15 journals from the Critical Care Medicine category, 1 of 15 from Cell and Tissue Engineering and 2 of 15 from Cell Biology provided a definition of misconduct.

Falsification as a form of misconduct was explicitly mentioned by 113 (28.3%) journals, fabrication by 104 (26.1%), plagiarism by 224 (56.1%), duplication by 242 (60.7%) and image manipulation by 154 (38.6%).

Procedures for responding to misconduct were described by 179 (44.9%) journals, including retraction, (n = 123; 30.8%) and expression of concern (n = 65; 16.3%), while 130 journals (32.6%) had other procedures for responding. The following categories had the highest number of journals with procedures for responding to misconduct: Medicine, Research and Experimental (10 of 15), Gastroenterology and Metabolism (10 of 15), Biotechnology and Applied Microbiology (10 of 15) and Hematology (12 of 15). In contrast, only 4 of 15 journals from Radiology, 2 of 15 from Cell and Tissue Engineering, 3 of 15 from Cardiac and Cardiovascular Systems, 2 of 15 from Critical Care Medicine, and 4 of 15 from Neurosciences had procedures for responding.

The use of plagiarism-checking services was declared by 112 (28.1%) journals, notably in Biotechnology and Applied Microbiology (8 of 15), Biochemistry and Molecular Biology (8 of 15), Obstetrics and Gynecology (8 of 15) and Critical Care Medicine (8 of 15). Infectious Disease (1 of 15), Psychiatry (1 of 15) and Gastroenterology and Metabolism (0 of 15) were the categories with the least use.

No significant differences in the IF were found between journals with and without a definition of misconduct (P = 0.074), including falsification (P = 0.096), plagiarism (P = 0.629), duplication (P = 0.415), use of plagiarism-checking service (P = 0.541) and procedures for responding (P = 0.136). There were significant differences with regard to fabrication (P = 0.016) and image manipulation (P = 0.006).

Comparison of journals that did (n = 239; 59.9%) or did not (n = 160; 40.1%) endorse policy-producing bodies’ guidelines showed that the former scored significantly higher than the latter in definition of misconduct (P<0.001), falsification (P<0.001), fabrication (P = 0.003), plagiarism (P<0.001), duplication (P<0.001), procedures for responding (P<0.001) including retraction (P = 0.002) and expression of concern (P<0.001), image manipulation (P<0.001) and plagiarism-checking service (P = 0.007) (Table 2). When considering the bodies separately, the differences for the presence of policies were significant for ICMJE, COPE and ORI, except for plagiarism-checking, where differences were significant for COPE only (P = 0.007).

**Table 2:** Prevalence (number and percentage) of misconduct policies and procedures for responding to misconduct allegations of journals that endorsed policies from editors’ associations, ORI or professional societies.

Policy	No endorsement (n = 160)	Endorsement (n = 239)	P
Mention of misconduct	96 (60.0)	183 (76.6)	<0.001
Definition of misconduct	33 (20.6)	107 (44.8)	<0.001
Falsification	23 (14.4)	90 (37.7)	<0.001
Fabrication	29 (18.1)	75 (31.4)	0.003
Plagiarism	63 (39.4)	161 (67.4)	<0.001
Duplication	77 (48.1)	165 (69.0)	<0.001
Other criteria of misconduct	16 (10.0)	53 (22.2)	0.002
Image manipulation	44 (27.8)	110 (46.2)	<0.001
Plagiarism-checking service	33 (20.6)	79 (33.1)	0.007
Procedures for responding	43 (27.0)	136 (57.1)	<0.001
Retraction	35 (22.0)	88 (36.8)	0.002
Expression of concern	8 (5.0)	57 (23.8)	<0.001

ORI denotes Office of Research Integrity.

Comparison of US and European journals showed no significant differences in any of the policies analyzed or in the generation of misconduct policies (journal, publisher or other sources). However, 48 (36.4%) European journals vs. 53 (24.1%) US journals used plagiarism-checking (P = 0.014) (Table 3).

**Table 3:** Prevalence (number and percentage) of misconduct policies and procedures for responding to misconduct allegations in journals from USA and Europe.

Policy	US journals (n = 220)	European journals (n = 132)	P
Mention of misconduct	158 (71.8)	94 (71.2)	0.903
Definition of misconduct	78 (35.5)	51 (38.6)	0.549
Falsification	67 (30.5)	37 (28.0)	0.629
Fabrication	68 (30.9)	31 (23.5)	0.134
Plagiarism	125 (56.8)	79 (59.8)	0.577
Duplication	135 (61.4)	85 (64.4)	0.570
Other criteria of misconduct	34 (15.5)	29 (22.0)	0.123
Image manipulation	96 (43.6)	48 (36.4)	0.168
Plagiarism-checking service	53 (24.1)	48 (36.4)	0.014
Procedures for responding	95 (43.2)	65 (49.2)	0.269
Retraction	68 (30.9)	40 (30.3)	0.905
Expression of concern	36 (16.4)	19 (14.4)	0.622

Ninety (22.6%) journals were published by Elsevier and 59 (14.8%) by Wiley-Blackwell (Table 1). Wiley-Blackwell scored significantly higher in definition of misconduct, falsification, fabrication and expression of concern but Elsevier scored higher in plagiarism-checking (Table 4).

**Table 4:** Prevalence (number and percentage) of misconduct policies and procedures for responding between journals published by Elsevier or Wiley-Blackwell.

Policy	Elsevier (n = 90)	Wiley-Blackwell (n = 59)	P
Mention of misconduct	69 (76.7)	42 (71.2)	0.453
Definition of misconduct	27 (30.0)	28 (47.5)	0.031
Falsification	11 (12.2)	26 (44.1)	<0.001
Fabrication	8 (8.9)	24 (40.7)	<0.001
Plagiarism	56 (62.2)	34 (57.6)	0.575
Duplication	60 (66.7)	36 (61.0)	0.481
Other criteria of misconduct	16 (17.8)	6 (10.2)	0.200
Image manipulation	31 (35.2)	29 (49.2)	0.092
Plagiarism-checking service	33 (36.7)	10 (16.9)	0.009
Procedures for responding	34 (37.8)	30 (51.7)	0.095
Retraction	25 (27.8)	25 (43.1)	0.054
Expression of concern	11 (12.2)	23 (39.7)	<0.001

Image manipulation was mentioned explicitly as misconduct or unethical by 154 (38.6%) journal websites, especially in the Medicine, Research and Experimental (13 of 15), Gastroenterology and Metabolism (12 of 15), Hematology (12 of 15) and Biochemistry and Molecular Biology (11 of 15) categories, but only by 1 journal from Infectious Diseases, 1 from Obstetrics and Gynecology, 2 from Critical Care Medicine, and 2 from Radiology. One hundred and ten (71.4%) of the 154 journals with an image manipulation policy vs. 128 (52.9%) of the 242 without a policy endorsed policy-producing bodies’ guidelines (P<0.001), with significant differences for ICMJE, COPE and ORI (Table 5).

**Table 5:** Number of journals (percentage) having or not an image manipulation policy according to endorsement of definitions and guidelines of editors’ associations, ORI and professional societies.

Source of policy	Image manipulation policy (n = 154)	No image manipulation policy (n = 242)	P
Any Policy-Producing Body	110 (71.4)	128 (52.9)	<0.001
ICMJE	76 (49.4)	82 (33.9)	0.002
COPE	82 (53.2)	74 (30.6)	<0.001
ORI	27 (17.5)	5 (2.1)	<0.001
WAME	21 (13.6)	23 (9.5)	0.202
CSE	4 (2.6)	2 (0.8)	0.213
Other bodies	11 (7.1)	10 (4.1)	0.192

ICMJE denotes International Committee of Medical Journal Editors; COPE, Committee on Publication Ethics; ORI, Office of Research Integrity; WAME, World Association of Medical Editors; CSE, Council of Science Editors.

As for the type of contents, 67.5% of 123 clinical vs. 45.7% of 162 basic journals subscribed to policy-producing bodies’ guidelines (p<0.001), with differences being significant for ICMJE (P<0.001). Regarding all the policies analyzed, 83 (51.2%) basic vs. 46 (37.4%) clinical journals had procedures for responding (P = 0.018), with differences being significant for retraction (P = 0.008) but not for expression of concern (P = 0.816). In addition, 67 (41.4%) basic vs. 38 (30.9%) clinical journals included image manipulation as a misconduct policy (P = 0.058). No significant differences were found for the remaining policies.

## Discussion

Our study comprehensively appraises misconduct policies of the top-ranked peer-reviewed biomedical journals and demonstrates that greater efforts are stilly required to raise the level of transparency and implementation of integrity procedures.

In deciding when to respond to allegations of misconduct, a definition of misconduct is essential [10]. Only 35.1% of journals provided explicit definitions of misconduct, and only 44.9% had procedures for responding to misconduct, including retraction (30.8%) and expression of concern (16.3%) and use of plagiarism-checking service (28.1%).

This is the first study to examine a large, comprehensive sample of top-ranked clinical and basic biomedical journals publishing original research. Although a 2006 study examined misconduct policies of biomedical journals with the highest IF (JCR, 2004), the sample was small (n = 50) and included 26 review-only journals, and found that only 7 journals had developed misconduct policies [11].

A 2009 study by Resnik *et al* on journal misconduct policies with a larger sample (n = 399) (JCR, 2008) analyzed a wider ranger of journals (including physical, engineering, and social science in addition to biomedical journals) through contact with editors (response rate of 49.4%), although the random sample was not representative of top-ranked journals [mean IF: 2.23 (SD: 3.05)], as it was in our study [12]. The authors found lower rates of policy development [47.7% had a formal (written) policy] than shown by our study, and lower rates of procedures (28.9% had a policy that only outlined procedures for handling misconduct) and definitions (15.7% had a policy that only defined misconduct). The journal IF was the only variable significantly associated with having a formal misconduct policy [12].

Another study by Resnik *et al* in 2010 examined the misconduct policies of social science journals [10], which were underrepresented in the 2009 study [12]. Combining the results with those of the previous study showed that, of the 350 journals (response rate of 43.8%) examined, 144 (41.1%) had formal misconduct policies and 206 (58.9%) did not. The journals studied had an average IF of 1.91. As in the 2009 study [12], only the journal IF was the only variable statistically associated with having a formal misconduct policy or not, with the scientific category not affecting the results [10].

A possible explanation for the differences in rates of policy development might be the differences in the IF of the journals included in our study and those of the studies by Resnik *et al* in 2009 [12] and 2010 [10].

We found significant differences according to the IF only when comparing journals mentioning fabrication and image manipulation as misconduct. Our results showed that duplication, plagiarism, and image manipulation seemed to be the misconduct items that mostly concern journals. The most prevalent misconduct policy was that for duplicate publications (60.7%). Although generally not considered a form of misconduct per se (for instance, COPE defines misconduct as “intention to cause others to regard as true that which is not true”), redundant or overlapping publication, often revealed by peer review, implies significant data republication with little original material added to previous work by the same authors [3], [13]. Duplicate publication, a subcategory of redundant publication, may be the easiest type misconduct to identify. It may nearly be classified as self-plagiarism, and can distort the literature, over-emphasizing the importance of a single study in meta-analyses [13].

Data showing a prevalence of duplicate publications of 8.5% in otolaryngology journals, many published within 12 months of the first article, prompted American editors of otolaryngology, head and neck surgery journals to coordinate responses to violations of publication ethics by sharing the name of the infractor and details of the infraction and, when necessary, suspending the author’s publishing privileges, thereby limiting attempts to resubmit the offending article to another journal [14]. The ICMJE advises editors to reject manuscripts where overlapping publication is detected and publish an editorial note detailing the infraction. The COPE suggests that authors’ institutions may be informed of the infraction [15].

A possible reason why duplicate publication was the most common matter addressed by misconduct policies in our study is that this is a legal and intellectual property issue for journals, as it may infringe copyrights. This may explain why so many journals ask authors if material has been published previously. Publishers are strongly motivated to prevent duplicate publication, and may be influencing policy in this way. The prevalence of duplicate publication may also be related to the specificity of health research and the influence of duplicate publications on systematic reviews and guidelines for practice. To explore this issue further, we analyzed whether the source of misconduct policies affected the prevalence of duplicate publication policies among journals with (n = 242) and without (n = 157) a duplication policy. When the policy was generated by the journal, only 22 (14.0%) journals did not have a duplication policy compared to 110 (45.5%) that did (P<0.001). Likewise, when the policy was generated by the publisher, only 9 (5.7%) journals did not have a duplication policy as compared to 134 (55.4%) that did (P<0.001) (results not shown).

We found that 56.1% of journals had adopted plagiarism policies and 28.1% claimed to use a plagiarism-checking service. Image manipulation was mentioned as misconduct by 38.6% journals but, surprisingly, by only 13.3% of Radiology journals. More basic (41.4%) than clinical (30.9%) journals mentioned image manipulation although the difference was not significant (P = 0.058). This may reflect the fact that basic science journals were the first to develop this policy [16].

Journals are increasingly screening papers for plagiarism and image manipulation to detect misconduct before publication [17]. Publicly-available software is routinely applied to submissions in some journals to detect any image manipulation [18]. Since October 2008, PLoS Medicine routinely checks articles for figure manipulation and states that if evidence is found of inappropriate manipulation they “reserve the right to ask for original data and if that is not satisfactory may decide not to accept the manuscript” [19]. Likewise, Diabetes and Diabetes Care adopted the policy of the Journal of Cell Biology on the manipulation of digital images in 2010, and uses imaging forensics software that scans all images and generates before and after reports on suspected manipulation [16], [18], [20].

In our study, misconduct policies were generated by the publisher in 35.8% of cases. Elsevier and Wiley-Blackwell had the most journals included (22.6% and 14.8%, respectively). Interestingly, only 16.9% of Wiley-Blackwell vs. 36.7% of Elsevier journals used a plagiarism-checking service (p = 0.009). However, a significantly higher proportion of Wiley-Blackwell journals included a definition of misconduct, falsification, fabrication and expression of concern among their policies. Wiley-Blackwell journals were also non-significantly better than Elsevier with respect to procedures for responding, retraction and image manipulation. Thus, Elsevier’s decision in 2008 to ask all its journals to become COPE members seems not to have had a significant impact on the adoption of misconduct policies by its scientific journals [21].

There were significant differences between journals subscribing or not to misconduct guidelines and definitions of editors’ associations, ORI or professional societies with respect to misconduct definition, falsification, fabrication, plagiarism, duplication, procedures for responding including retraction and expression of concern, image manipulation, and plagiarism-checking service. Considering the bodies separately, the differences for all these variables were significant for ICMJE, COPE and ORI, except for plagiarism-checking, where differences were significant for COPE only. Procedures for responding to misconduct were stated by 44.9% of journals, including 51.2% of basic and 37.4% of clinical journals (p = 0.018), with differences being significant for retraction but not for expression of concern. Only 30.8% of journals included formally implemented retraction and 16.3% expression of concern among responding procedures.

The number of retractions has increased markedly in the last decade, especially those concerning misconduct, with the rise in retractions being higher than the rise in the number of published articles (see Table 6) [22]. Although retraction is a serious and, in some cases, career threatening measure, evidence suggests that the response of journals to serious misconduct findings affecting their publications is not consistent [23]. The lack of the necessary investigative resources, definitions of misconduct, investigative procedures and policies, allied to a lack of experience in such matters and of currently accepted definitions of misconduct, may explain why few editors seem willing to undertake such investigations [3]. Concerns about legal action by authors or the belief that only authors should make retractions may also explain editors’ and publishers’ reluctance to take action.

**Table 6:** Number of retractions and published articles listed in PubMed since 1970*.

Year	Number of retractions	Number of items indexed	Retractions per 1,000 items (%)
2010–2012	294	2,385,130	12.33
2000–2009	1482	6,468,664	22.91
1990–1999	349	4,390,650	7.95
1980–1989	131	3,299,814	3.97
1970–1979	25	2,447,305	1.02

* Search performed on August 28, 2012.

Unfortunately, although retractions may be triggered both by genuine mistakes and by misconduct, the reasons for retraction are not always stated [23]. Regrettably, the National Library of Medicine does not indicate whether manuscripts are withdrawn due to true mistakes or to possible misconduct [3]. The 2009 COPE retraction guidelines [24] and the ICMJE [25] recommend indicating the reason for retraction, avoiding stigmatization of responsible authors who notify journals of possible problems with their study.

A recent study by Resnik and Dinse analyzed retractions or corrections in papers associated with official findings of misconduct by evaluating all 208 resolved cases containing official determinations of research misconduct reported by the ORI between 1992 and 2011 [26]. The aim was to analyze how often authors stated that ethical problems existed in associated articles in notices of retraction or correction. The authors evaluated 119 articles subject to detailed published correction or retraction and found that the issued stated that misconduct or other ethical problems were the motive for the retraction or correction in only 41.2% of cases, with only 32.8% specifically stating the ethical problem, e.g., plagiarism, fabrication, or falsification. In the remaining 58.8% of cases, the stated reason given for retraction or correction was data loss, failure to reproduce the results or simple error, rather than misconduct that was the real reason for correction or retraction. In fact, for 7.8% of retracted articles, there was only a notice of retraction without more explanation. The authors concluded that authors retracting or correcting papers for reasons of misconduct are often not providing truthful explanations of the reasons behind these actions [26]. This could be seen as a policy concern for journals that may not be completely transparent about the reasons for retractions or corrections.

A comprehensive review by Fang *et al* recently showed that misconduct may be more pervasive than previously thought [27]. They evaluated all 2,047 retracted biomedical and life-science research articles indexed by PubMed by May, 3, 2012 and found that the retraction could be attributed to error in only 21.3% of cases and to misconduct in 67.4% of cases, which included suspected or actual fraud (43.4%), duplicate publication (14.2%), and plagiarism (9.8%). Although the authors found a 10-fold rise in the percentage of articles due to fraud since 1975, they suggest that retraction statements that lack the necessary detail or are downright misleading may have resulted in the true extent of fraud in scientific publications being seriously underestimated.

Non-retraction of articles containing false information may have consequences. Even officially-retracted articles are included as citations and mentioned in other studies. Investigation of misconduct is time-consuming and may fail, even after the identification of fraud [1.28]. In the case of drug trials, this could mean the continuation of therapy based on misinformation for long periods before retraction of an article is widely disseminated [29]–[31].

Our study shows that a high proportion of journals have not implemented misconduct policies. Although some policies might have been missed when journal websites were reviewed in December 2011, websites provide the main permanent source of editorial and publishing policies, and should include ethical requirements for submitted manuscripts and misconduct policies. Even when present, ethical statements and requirements are commonly placed in different sections, making identification difficult unless a specific search is made. Only instructions for authors and submission guidelines are commonly placed in high-visibility sites.

Many authors remain unaware of publication guidelines or pay them little heed, despite the possible consequences if ethical infringements are discovered [32]. Some professional societies and their journals have specific detailed guidelines for ethical conduct and retractions in the instructions to authors, including American Society for Microbiology journals such as Infection and Immunity [33], the American Heart Association [34] and the American Headache Society, whose journal Headache even has a policy on redundant publication [13]. The same is true for image manipulation, where, for instance, the PLoS Medicine website provides examples of inappropriate manipulation in its figure guidelines [19].

Theoretically, responsible researchers would not engage in the misconduct behaviors discussed here, even without explicit misconduct statements and policies. Unfortunately, the prevalence of research misconduct seems to be higher than might be expected [35], [36]. A meta-analysis of surveys of misconduct experiences found that about 2% of scientists admitted fabricating, falsifying or modifying data or results at least once and up to one third admitted other questionable research practices including “changing the design, methodology or results of a study in response to pressures from a funding source” [35]. In surveys of the behavior of colleagues, fabrication, falsification and modification had been observed by over 14% of respondents and other questionable practices by up to 72% [35].

We recommend that ethical guidelines in publishing, including misconduct policies and procedures for responding, be easily accessible (i.e., using the minimum number of clicks to get from the home page to the policy guidelines), and are placed in highly-visible sites and similar locations to ensure that authors know the conduct they must abide by and the consequences of not doing so. Just as authors rely upon instructions to authors to write their research findings, the ethical guidelines should be an essential tool for addressing research integrity topics including misconduct policies, procedures for responding, financial and non-financial competing interests, and authorship issues. The current variability in location and lack of visibility meant that the authors of our study who searched the journals’ websites spent significant amounts of time locating the relevant policies, especially at the beginning, when they were less accustomed to the task. Although not analyzed, we observed that misconduct policies were placed on web pages such as ‘Policies’, ‘Editorial Policies’, ‘Submit your manuscript’ or ‘About this journal’. In some cases, journals using policies generated by publishers or editorial associations simply put the link, assuming that any ‘interested authors’ will find the policies by clicking on the link. Finally, in the same way that many journals require signed conflicts of interest and authorship forms before acceptance or upon submission, we suggest that authors sign a specifically-designed, comprehensive ethics form that explicitly covers the issues described by our study, and not merely a general ethical statement.

The study limitations include the cross-sectional design and selection of journals. Data was obtained entirely from journal websites, and some policies might have been missed during the examination. In addition, this is primarily a descriptive study and it is unclear what the impact of misconduct policies might be, for example, whether there is any association between misconduct policies and the prevalence of misconduct or the ability to mitigate it. Nevertheless, this survey may be a starting point for more transparency on how misconduct policies are implemented by journals. Just as transparent criteria for authorship are key in guaranteeing untainted scientific investigation and aid readers to decide the type of contribution made by each author [37], journals that fail to post explicit policies on misconduct are doing science a disservice because, without unequivocal support from scientific journals, a reduction in fraudulent research conduct is unlikely.

In conclusion, about one-third of journals provided explicit definitions of misconduct and less than half had procedures for responding. Duplication, plagiarism and image manipulation were the misconduct items scoring highest. There were significant differences in policies and procedures between publishers. Endorsing policy-producing bodies’ guidelines and definitions was positively associated with implementation of policies and procedures. Journals and their publishers should pursue consensus and standardize their policies globally and actively in order to increase public trust in the integrity of the published record in biomedicine.

## Supporting Information

Table S1 **Journals included according to medical category and impact factor (2010).**
(DOCX)

Appendix S1 **Data Abstraction Form.**
(DOCX)

## References

[1] Wager E (2011) Coping with scientific misconduct. BMJ 343: d6586. 22016451 10.1136/bmj.d6586

[2] Definition of research misconduct. Office of Research Integrity. US Department of Health & Human Services. Available: http://ori.hhs.gov/definition-misconduct. Accessed 2012 October 22.

[3] Council of Science Editors’ White Paper on Promoting Integrity in Scientific Journal Publications (2012 Update). Available: http://www.councilscienceeditors.org/i4a/pages/index.cfm?pageid=333.1. Accessed 2012 September 2.

[4] Graf C, Wager E, Bowman A, Fiack S, Scott-Lichter D, et al (2007) Best practice guidelines on publication ethics: A publisher’s perspective. Int J Clin Pract 61: 1–26. 17206953 10.1111/j.1742-1241.2006.01230.x PMC1804120

[5] Managing allegations of scientific misconduct: A Guidance Document for Editors. January 2000. Office of Research Integrity. Office of Public Health and Science. US Department of Health & Human Services. Available: http://ori.hhs.gov/content/handbooks-and-guidelines. Accessed 2012 October 22.

[6] Scheetz MD (2000) Instructions to the Author: An Integrity Issue. Investigating Research Integrity. Proceedings of the First ORI Research Conference on Research Integrity; Nov 19–20. Bethesda, Maryland: Office of Research Integrity, US Department of Health & Human Services 2001: 285–290.

[7] Drazen JM, Van der Weyden MB, Sahni P, Rosenberg J, Marusic A, et al (2009) Uniform format for disclosure of competing interests in ICMJE journals. N Engl J Med 361: 1896–1897. 19825973 10.1056/NEJMe0909052

[8] Journal Citation Reports. Thomson Reuters. Available: http://thomsonreuters.com/products_services/science/science_products/az/journal_citation_reports. Accessed 2012 September 2.

[9] Steneck NH (2006) Fostering integrity in research: definitions, current knowledge, and future directions. Sci Eng Ethics 12: 53–74. 16501647 10.1007/pl00022268

[10] Resnik DB, Patrone D, Peddada S (2010) Research misconduct policies of social science journals and impact factor. Account Res 17: 79–84. 20306350 10.1080/08989621003641181 PMC3065865

[11] Redman BK, Merz JF (2006) Research misconduct policies of high impact biomedical journals. Account Res 13: 247–258. 17124760 10.1080/08989620600848199

[12] Resnik DB, Peddada S, Brunson W (2009) Research misconduct policies of scientific journals. Account Res 16: 254–267. 19757231 10.1080/08989620903190299 PMC3943876

[13] Roberts J (2009) An author’s guide to publication ethics: a review of emerging standards in biomedical journals. Headache 49: 578–589. 19338617 10.1111/j.1526-4610.2009.01379.x

[14] Benninger MS, Jackler RK, Johns MM, Johnson JT, Kennedy DW, et al (2005) Consortium of otolaryngology–head and neck surgery journals to collaborate in maintenance of high ethical standards. Arch Otolaryngol Head Neck Surg 131: 381–382. 15897414 10.1001/archotol.131.5.381

[15] COPE flowcharts. What to do if you suspect redundant (duplicate) publication. 16. Available: http://publicationethics.org/resources/flowcharts. Accessed 2012 September 2.

[16] Rossner M, Yamada KM (2004) What’s in a picture? The temptation of image manipulation. J Cell Biol 166: 11–15. 15240566 10.1083/jcb.200406019 PMC2172141

[17] Wager E, Kleinert S (2012) Cooperation between research institutions and journals on research integrity cases: guidance from the Committee on Publication Ethics (COPE). Maturitas 72: 165–169. 22541357 10.1016/j.maturitas.2012.03.011

[18] Diabetes. Publication Policies and Procedures (2011) Available: http://diabetes.diabetesjournals.org/site/misc/ifora.xhtml.Accessed 2012 September 2.

[19] PLoS Medicine. Guidelines for Figure and Table Preparation. Available: http://www.plosmedicine.org/static/figureGuidelines.action#manipulation. Accessed 2012 September 2.

[20] Kohler CS; Publications Policy Committee of the American Diabetes Association. Updates to policies and procedures related to potential scientific and academic misconduct in the journals of the American Diabetes Association (2012) Diabetes. 61: 38–39. 10.2337/db11-1432 PMC3237643 22187374

[21] COPE announcement: All Elsevier journals become COPE members. Editors will benefit from new partnership (13 February 2008). Available: http://publicationethics.org/newsevents/all-elsevier-journals-become-cope-members. Accessed 2012 September 2.

[22] Editorial (2011) Reaction to retractions. Nat Med 17: 1523. 22146429 10.1038/nm.2611

[23] Wager E, Williams P (2011) Why and how do journals retract articles? An analysis of Medline retractions 1988–2008. J Med Ethics 37: 567–570. 21486985 10.1136/jme.2010.040964

[24] COPE’s retraction guidelines (2009). Available: http://publicationethics.org/newsevents/cope%E2%80%99s-retraction-guidelines.Accessed 2012 September 2.

[25] International Committee of Medical Journal Editors. Uniform Requirements for Manuscripts Submitted to Biomedical Journals: Publishing and Editorial Issues Related to Publication in Biomedical Journals: Corrections, Retractions and “Expressions of Concern”. Available: http://www.icmje.org/publishing_2corrections.html. Accessed 2012 September 2.

[26] Resnik DB, Dinse GE (2012) Scientific retractions and corrections related to misconduct findings. J Med Ethics doi: 10.1136/medethics-2012-100766. 10.1136/medethics-2012-100766 PMC3525741 22942373

[27] Fang FC, Steen RG, Casadevall A (2012) Misconduct accounts for the majority of retracted scientific publications. Proc Natl Acad Sci U S A 109: 17028–17033. 23027971 10.1073/pnas.1212247109 PMC3479492

[28] Sox HC, Rennie D (2006) Research misconduct, retraction, and cleansing the medical literature: Lessons from the Poehlman case. Ann Intern Med 144: 609–613. 16522625 10.7326/0003-4819-144-8-200604180-00123

[29] Hall RI (2012) Mea culpa: scientific misconduct. J Cardiothorac Vasc Anesth 26: 181–185. 22257830 10.1053/j.jvca.2011.12.003

[30] Trikalinos NA, Evangelou E, Ioannidis JP (2008) Falsified papers in high-impact journals were slow to retract and indistinguishable from nonfraudulent papers. J Clin Epidemiol 61: 464–470. 18394539 10.1016/j.jclinepi.2007.11.019 PMC4699806

[31] Steen RG (2011) Retractions in the medical literature: How many patients are put at risk by flawed research? J Med Ethics 37: 688–692. 21586404 10.1136/jme.2011.043133

[32] Barrett KA, Funk CL, Macrina FL (2005) Awareness of publication guidelines and the responsible conduct of research. Account Res 12: 193–206. 16634171 10.1080/08989620500217321

[33] Fang FC, Casadevall A (2011) Retracted science and the retraction index. Infect Immun 79: 3855–3859. 21825063 10.1128/IAI.05661-11 PMC3187237

[34] Miano JM (2010) What is truth? Standards of scientific integrity in American Heart Association journals. Arterioscler Thromb Vasc Biol 30: 1–4. 20018938 10.1161/ATVBAHA.109.200204

[35] Fanelli D (2009) How many scientists fabricate and falsify research? A systematic review and meta-analysis of survey data. PLoS ONE 4: e5738. 19478950 10.1371/journal.pone.0005738 PMC2685008

[36] Martinson BC, Anderson MS, de Vries R (2005) Scientists behaving badly. Nature 435: 737–738. 15944677 10.1038/435737a

[37] Bosch X, Pericas JM, Hernández C, Torrents A (2012) A comparison of authorship policies at top-ranked peer-reviewed biomedical journals. Arch Intern Med 172: 70–72. 22232152 10.1001/archinternmed.2011.600

