# Supplementary material for: Misconduct Policies in High-Impact Biomedical Journals
Source: PLoS One. 2012 Dec 19;7(12):e51928. doi: 10.1371/journal.pone.0051928 (PMC3526485; doi:10.1371/journal.pone.0051928)
Supplement: Table S1 — Journals included according to medical category and impact factor (2010). (DOCX) [file pone.0051928.s001.docx]

**Table S1**. Journals included according to medical category and impact factor (2010)

|  | Journal name | Medical category |
| --- | --- | --- |
| 1 | Circulation | Cardiac and Cardiovascular Systems |
| 2 | J Am Coll Cardiol | Cardiac and Cardiovascular Systems |
| 3 | Eur Heart J | Cardiac and Cardiovascular Systems |
| 4 | Circ Res | Cardiac and Cardiovascular Systems |
| 5 | Int J Cardiol | Cardiac and Cardiovascular Systems |
| 6 | Nat Clin Pract Card | Cardiac and Cardiovascular Systems |
| 7 | Basic Res Cardiol | Cardiac and Cardiovascular Systems |
| 8 | Cardiovasc Res | Cardiac and Cardiovascular Systems |
| 9 | JACC-Cardiovasc Inte | Cardiac and Cardiovascular Systems |
| 10 | J Mol Cel Cardiol | Cardiac and Cardiovascular Systems |
| 11 | Am Heart J | Cardiac and Cardiovascular Systems |
| 12 | Circ-Cardiovasc Qual | Cardiac and Cardiovascular Systems |
| 13 | Circ-Arrhythmia Elec | Cardiac and Cardiovascular Systems |
| 14 | Circ-Cardiovasc Imag | Cardiac and Cardiovascular Systems |
| 15 | Heart | Cardiac and Cardiovascular Systems |
| 16 | Am J Resp Crit Care | Critical Care Medicine |
| 17 | Crit Care Med | Critical Care Medicine |
| 18 | Intens Care Med | Critical Care Medicine |
| 19 | Crit Care | Critical Care Medicine |
| 20 | Resuscitation | Critical Care Medicine |
| 21 | J Neurotrauma | Critical Care Medicine |
| 22 | Shock | Critical Care Medicine |
| 23 | J Trauma | Critical Care Medicine |
| 24 | Pediatr Crit Care Med | Critical Care Medicine |
| 25 | Neurocritical Care | Critical Care Medicine |
| 26 | Injury | Critical Care Medicine |
| 27 | J Crit Care | Critical Care Medicine |
| 28 | Burns | Critical Care Medicine |
| 29 | Am J Crit Care | Critical Care Medicine |
| 30 | Resp Care | Critical Care Medicine |
| 31 | Cell Metab | Endocrinology and Metabolism |
| 32 | Diabetes | Endocrinology and Metabolism |
| 33 | Antioxid Perox Sign | Endocrinology and Metabolism |
| 34 | Diabetes Care | Endocrinology and Metabolism |
| 35 | J Bone Miner Res | Endocrinology and Metabolism |
| 36 | Diabetologia | Endocrinology and Metabolism |
| 37 | J Clin Endocr Metab | Endocrinology and Metabolism |
| 38 | Free Radical Bio Med | Endocrinology and Metabolism |
| 39 | Psychoneuroendocrino | Endocrinology and Metabolism |
| 40 | Int J Obesity | Endocrinology and Metabolism |
| 41 | Endocrinology | Endocrinology and Metabolism |
| 42 | Mol Endocrinol | Endocrinology and Metabolism |
| 43 | Osteoporosis Int | Endocrinology and Metabolism |
| 44 | J Neuroendocrinol | Endocrinology and Metabolism |
| 45 | Bone | Endocrinology and Metabolism |
| 46 | Gastroenterology | Gastroenterology and Hepatology |
| 47 | Hepatology | Gastroenterology and Hepatology |
| 48 | Gut | Gastroenterology and Hepatology |
| 49 | J Hepatol | Gastroenterology and Hepatology |
| 50 | Am J Gastroenterol | Gastroenterology and Hepatology |
| 51 | Endoscopy | Gastroenterology and Hepatology |
| 52 | Gastrointestinal endosc | Gastroenterology and Hepatology |
| 53 | Clin Gastroenterol H | Gastroenterology and Hepatology |
| 54 | Inflamm Bowel Dis | Gastroenterology and Hepatology |
| 55 | Aliment Pharm Ther | Gastroenterology and Hepatology |
| 56 | Liver Int | Gastroenterology and Hepatology |
| 57 | J Gastroenterol | Gastroenterology and Hepatology |
| 58 | J Viral Hepatitis | Gastroenterology and Hepatology |
| 59 | Neurogastroent Motil | Gastroenterology and Hepatology |
| 60 | Helicobacter | Gastroenterology and Hepatology |
| 61 | Aging Cell | Geriatrics and Gerontology |
| 62 | Neuro Biol | Geriatrics and Gerontology |
| 63 | Age | Geriatrics and Gerontology |
| 64 | Mech Ageing Dev | Geriatrics and Gerontology |
| 65 | J Am Med Dir Assoc | Geriatrics and Gerontology |
| 66 | Rejuv Res | Geriatrics and Gerontology |
| 67 | J Gerontol A-Biol | Geriatrics and Gerontology |
| 68 | J Am Geriatr Soc | Geriatrics and Gerontology |
| 69 | Exp Gerontol | Geriatrics and Gerontology |
| 70 | Am J Geriat Psychiat | Geriatrics and Gerontology |
| 71 | Biogerontology | Geriatrics and Gerontology |
| 72 | Age Ageing | Geriatrics and Gerontology |
| 73 | Drug Aging | Geriatrics and Gerontology |
| 74 | J Nutr | Geriatrics and Gerontology |
| 75 | Int Psychogeriatr | Geriatrics and Gerontology |
| 76 | Blood | Hematology |
| 77 | Leukemia | Hematology |
| 78 | Arterioscl Throm Vas | Hematology |
| 79 | Haematol-Hematol J | Hematology |
| 80 | J Thromb Haemost | Hematology |
| 81 | Brit J Haematol | Hematology |
| 82 | Thromb Haemostasis | Hematology |
| 83 | J Leukocyte Biol | Hematology |
| 84 | J Cerebr Blood F Met | Hematology |
| 85 | Bone Marrow Transpl | Hematology |
| 86 | Am J Hematol | Hematology |
| 87 | Transfusion | Hematology |
| 88 | Vox Sang | Hematology |
| 89 | Biol Blood Marrow Tr | Hematology |
| 90 | Exp Hematol | Hematology |
| 91 | Lancet Infect Dis | Infectious Diseases |
| 92 | Clin Infect Dis | Infectious Diseases |
| 93 | Emerg Infect Dis | Infectious Diseases |
| 94 | AIDS | Infectious Diseases |
| 95 | Clin Microbiol Infect | Infectious Diseases |
| 96 | J Antimicrob Chemoth | Infectious Diseases |
| 97 | JAIDS | Infectious Diseases |
| 98 | Infect Immun | Infectious Diseases |
| 99 | Influenza Other Resp | Infectious Diseases |
| 100 | J Infect | Infectious Diseases |
| 101 | Antivir Ther | Infectious Diseases |
| 102 | Infect Cont Hosp Ep | Infectious Diseases |
| 103 | HIV Med | Infectious Diseases |
| 104 | J Viral Hepatitis | Infectious Diseases |
| 105 | Sex Transm Dis | Infectious Diseases |
| 106 | NEJM | Medicine, General and Internal |
| 107 | Lancet | Medicine, General and Internal |
| 108 | JAMA | Medicine, General and Internal |
| 109 | Ann Intern Med | Medicine, General and Internal |
| 110 | Plos Med | Medicine, General and Internal |
| 111 | Brit Med J | Medicine, General and Internal |
| 112 | Arch Intern Med | Medicine, General and Internal |
| 113 | Can Med Assoc J | Medicine, General and Internal |
| 114 | J Intern Med | Medicine, General and Internal |
| 115 | BMC Med | Medicine, General and Internal |
| 116 | Mayo Clin Proc | Medicine, General and Internal |
| 117 | Am J Med | Medicine, General and Internal |
| 118 | Ann Fam Med | Medicine, General and Internal |
| 119 | Ann Med | Medicine, General and Internal |
| 120 | Am J Prev Med | Medicine, General and Internal |
| 121 | Behav Brain Sci | Neurosciences |
| 122 | Nat Neurosci | Neurosciences |
| 123 | Neuron | Neurosciences |
| 124 | Ann Neurol | Neurosciences |
| 125 | Brain | Neurosciences |
| 126 | Acta Neuropathol | Neurosciences |
| 127 | J Neurosci | Neurosciences |
| 128 | Cortex | Neurosciences |
| 129 | Cereb Cortex | Neurosciences |
| 130 | Neurobiol Aging | Neurosciences |
| 131 | Neurotherapeutics | Neurosciences |
| 132 | J Neuroinflamm | Neurosciences |
| 133 | Sleep | Neurosciences |
| 134 | Mol Neurodegener | Neurosciences |
| 135 | J Cognitive Neurosci | Neurosciences |
| 136 | Obstet Gynecol | Obstetrics and Gynecology |
| 137 | Hum Reprod | Obstetrics and Gynecology |
| 138 | Gynecol Oncol | Obstetrics and Gynecology |
| 139 | BJOG-Int J Obstet Gy | Obstetrics and Gynecology |
| 140 | Menopause | Obstetrics and Gynecology |
| 141 | Am J Obstet Gynecol | Obstetrics and Gynecology |
| 142 | Ultrasound Obstet Gynecol | Obstetrics and Gynecology |
| 143 | Fertil Steril | Obstetrics and Gynecology |
| 144 | Placenta | Obstetrics and Gynecology |
| 145 | Reprod Sci | Obstetrics and Gynecology |
| 146 | Contraception | Obstetrics and Gynecology |
| 147 | Int J Urogynecol J | Obstetrics and Gynecology |
| 148 | Maturitas | Obstetrics and Gynecology |
| 149 | Reprod Biomed Online | Obstetrics and Gynecology |
| 150 | Prenatal Diag | Obstetrics and Gynecology |
| 151 | Cancer Cell | Oncology |
| 152 | J Clin Oncol | Oncology |
| 153 | Lancet Oncol | Oncology |
| 154 | J Natl Cancer I | Oncology |
| 155 | EJC Suppl | Oncology |
| 156 | Cancer Research | Oncology |
| 157 | Clin Cancer Res | Oncology |
| 158 | J Pathol | Oncology |
| 159 | Ann Oncol | Oncology |
| 160 | Oncologist | Oncology |
| 161 | Breast Cancer Res | Oncology |
| 162 | Neuro-Oncology | Oncology |
| 163 | Neoplasia | Oncology |
| 164 | Carcinogenesis | Oncology |
| 165 | Mol Cancer Ther | Oncology |
| 166 | Pediatrics | Pediatrics |
| 167 | J Am Acad Child Psy | Pediatrics |
| 168 | J Pediatr-US | Pediatrics |
| 169 | Arch Pediatr Adol Med | Pediatrics |
| 170 | Dev Med Child Neurol | Pediatrics |
| 171 | Arch Dis-Child Fetal | Pediatrics |
| 172 | J Adolescent Health | Pediatrics |
| 173 | Pediatr Infect Dis | Pediatrics |
| 174 | Pediatr Allerg Imm-UK | Pediatrics |
| 175 | Pediatr Res | Pediatrics |
| 176 | Int J Pediatr Obes | Pediatrics |
| 177 | Arch Dis Child | Pediatrics |
| 178 | Acad Pediatr | Pediatrics |
| 179 | J Child Adol Psychop | Pediatrics |
| 180 | Matern Child Nutric | Pediatrics |
| 181 | Mol Psychiatr | Psychiatry |
| 182 | Am J Psychiat | Psychiatry |
| 183 | Arch Gen Psychiat | Psychiatry |
| 184 | Biol Psychiat | Psychiatry |
| 185 | Schizofrenia Bull | Psychiatry |
| 186 | Neuropsychopharmacol | Psychiatry |
| 187 | Psychoter Psychosom | Psychiatry |
| 188 | Brit J Psychiat | Psychiatry |
| 189 | World Psychiatry | Psychiatry |
| 190 | Bipolar Disord | Psychiatry |
| 191 | Psychol Med | Psychiatry |
| 192 | J Clin Psychiat | Psychiatry |
| 193 | J Psychiatr Neurosci | Psychiatry |
| 194 | J Clin Psychopharm | Psychiatry |
| 195 | J Neurol Neurosur Ps | Psychiatry |
| 196 | Thorax | Respiratory System |
| 197 | Chest | Respiratory System |
| 198 | Eur Respir J | Respiratory System |
| 199 | Am J Resp Cell Mol | Respiratory System |
| 200 | Am J Physiol-Lung C | Respiratory System |
| 201 | J Thorac Oncol | Respiratory System |
| 202 | Ann Thorac Surg | Respiratory System |
| 203 | J Thorac Cardiov Sur | Respiratory System |
| 204 | J Heart Lung Transpl | Respiratory System |
| 205 | Lung Cancer | Respiratory System |
| 206 | Resp Res | Respiratory System |
| 207 | J Cyst Fibros | Respiratory System |
| 208 | Tuberculosis | Respiratory System |
| 209 | Int J Tuberc Lung D | Respiratory System |
| 210 | Respiration | Respiratory System |
| 211 | Ann Rheum Dis | Rheumatology |
| 212 | Arthritis Rheum-US | Rheumatology |
| 213 | Anthrit Care Res | Rheumatology |
| 214 | Arthritis Res Ther | Rheumatology |
| 215 | Rheumatology | Rheumatology |
| 216 | Osteoarthr Cartilage | Rheumatology |
| 217 | J Rheumatol | Rheumatology |
| 218 | Lupus | Rheumatology |
| 219 | Scand J Rheumatol | Rheumatology |
| 220 | Joint Bone Spine | Rheumatology |
| 221 | Clin Exp Rheumatol | Rheumatology |
| 222 | BMC Musculoeskeletal Dis | Rheumatology |
| 223 | Mod Rheumatol | Rheumatology |
| 224 | Clin Rheumatol | Rheumatology |
| 225 | Rheumatol Int | Rheumatology |
| 226 | Eur Urol | Urology and Nephrology |
| 227 | J Am Soc Nephrol | Urology and Nephrology |
| 228 | Kidney Int | Urology and Nephrology |
| 229 | Am J Kidney Dis | Urology and Nephrology |
| 230 | Clin J Am Soc Nephro | Urology and Nephrology |
| 231 | J Sex Med | Urology and Nephrology |
| 232 | J Urology | Urology and Nephrology |
| 233 | Am J Physiol-renal | Urology and Nephrology |
| 234 | Nephrol Dial Transpl | Urology and Nephrology |
| 235 | Prostate | Urology and Nephrology |
| 236 | BJU Int | Urology and Nephrology |
| 237 | Urol Oncol-Semin Or | Urology and Nephrology |
| 238 | Neurourol Urodynam | Urology and Nephrology |
| 239 | Nephron Exp Nephrol | Urology and Nephrology |
| 240 | Am J Nephrol | Urology and Nephrology |
| 241 | Nat Chem Biol | Biochemistry and Molecular Biology |
| 242 | Nat Struct Mol Biol | Biochemistry and Molecular Biology |
| 243 | Embo J | Biochemistry and Molecular Biology |
| 244 | Curr Biol | Biochemistry and Molecular Biology |
| 245 | Mol Syst Biol | Biochemistry and Molecular Biology |
| 246 | Eur Cells Mater | Biochemistry and Molecular Biology |
| 247 | Plant Cell | Biochemistry and Molecular Biology |
| 248 | Cell Death Differ | Biochemistry and Molecular Biology |
| 249 | Nucleic acid res | Biochemistry and Molecular Biology |
| 250 | Embo Rep | Biochemistry and Molecular Biology |
| 251 | Mol Ecol | Biochemistry and Molecular Biology |
| 252 | Structure | Biochemistry and Molecular Biology |
| 253 | Acta Crystallogr D | Biochemistry and Molecular Biology |
| 254 | Mol Cell Biol | Biochemistry and Molecular Biology |
| 255 | J Lipid Res | Biochemistry and Molecular Biology |
| 256 | Plos Biol | Biology |
| 257 | Faseb J | Biology |
| 258 | Philos TR Soc B | Biology |
| 259 | Bioscience | Biology |
| 260 | BMC Biol | Biology |
| 261 | P Roy Soc B-Biol Sci | Biology |
| 262 | Plos One | Biology |
| 263 | Biol Direct | Biology |
| 264 | Biol Letters | Biology |
| 265 | Bioelectrochemistry | Biology |
| 266 | J Biol Rhythm | Biology |
| 267 | Geobiology | Biology |
| 268 | J Exp Biol | Biology |
| 269 | J Math Biol | Biology |
| 270 | Cell | Cell Biology |
| 271 | Nat Cell Bio | Cell Biology |
| 272 | Moll Cell | Cell Biology |
| 273 | Dev Cell | Cell Biology |
| 274 | J Moll Cell Biol | Cell Biology |
| 275 | J Cell Biol | Cell Biology |
| 276 | Cell Res | Cell Biology |
| 277 | Cell Mol Life Sci | Cell Biology |
| 278 | Autophagy | Cell Biology |
| 279 | Faseb J | Cell Biology |
| 280 | Sci Signal | Cell Biology |
| 281 | Structure | Cell Biology |
| 282 | J Cell Sci | Cell Biology |
| 283 | Mol Cell Biol | Cell Biology |
| 284 | Mol Biol Cell | Cell Biology |
| 285 | Cell Stem Cell | Cell and Tissue Engineering |
| 286 | Cell Transpl | Cell and Tissue Engineering |
| 287 | Stem Cells Dev | Cell and Tissue Engineering |
| 288 | Tissue Eng | Cell and Tissue Engineering |
| 289 | J Tissue Eng Regen M | Cell and Tissue Engineering |
| 290 | Stem Cell Res | Cell and Tissue Engineering |
| 291 | Regen Med | Cell and Tissue Engineering |
| 292 | Cytotherapy | Cell and Tissue Engineering |
| 293 | Cell Repr | Cell and Tissue Engineering |
| 294 | Cell Mol Bio Eng | Cell and Tissue Engineering |
| 295 | Nat Genet | Genetics and Heredity |
| 296 | Genome Res | Genetics and Heredity |
| 297 | Gene Dev | Genetics and Heredity |
| 298 | Am J Hum Genet | Genetics and Heredity |
| 299 | Plos Genet | Genetics and Heredity |
| 300 | Hum Mol Genet | Genetics and Heredity |
| 301 | Oncogene | Genetics and Heredity |
| 302 | J Med Genet | Genetics and Heredity |
| 303 | Hum Mutat | Genetics and Heredity |
| 304 | Evolution | Genetics and Heredity |
| 305 | Mol Biol Evol | Genetics and Heredity |
| 306 | Genet Med | Genetics and Heredity |
| 307 | Hum Genet | Genetics and Heredity |
| 308 | DNA res | Genetics and Heredity |
| 309 | Epigenet Chromatin | Genetics and Heredity |
| 310 | Nat Immunol | Immunology |
| 311 | Immunity | Immunology |
| 312 | J Allergy Clin Immun | Immunology |
| 313 | J Autoimmun | Immunology |
| 314 | Mucosal Immun | Immunology |
| 315 | Allergy | Immunology |
| 316 | J Immunol | Immunology |
| 317 | Europ J Immunol | Immunology |
| 318 | Genes Immun | Immunology |
| 319 | Cancer Immunol Immun | Immunology |
| 320 | Clin Exp Allergy | Immunology |
| 321 | Biodrugs | Immunology |
| 322 | Immunobiology | Immunology |
| 323 | Eur J Inflamm | Immunology |
| 324 | Brain Behav Immun | Immunology |
| 325 | Nat Med | Medicine, Research and Experimental |
| 326 | J Exp Med | Medicine, Research and Experimental |
| 327 | J Clin Invest | Medicine, Research and Experimental |
| 328 | Mol Aspects Med | Medicine, Research and Experimental |
| 329 | EMBO Mol Med | Medicine, Research and Experimental |
| 330 | Mol Med | Medicine, Research and Experimental |
| 331 | J Mol Med | Medicine, Research and Experimental |
| 332 | Nanomed-Nanotechnol | Medicine, Research and Experimental |
| 333 | Hum Gene Ther | Medicine, Research and Experimental |
| 334 | Clin Sci | Medicine, Research and Experimental |
| 335 | J Cell Mol Med | Medicine, Research and Experimental |
| 336 | Gene Ther | Medicine, Research and Experimental |
| 337 | ALTEX-Altern Anim Ex | Medicine, Research and Experimental |
| 338 | Lab Invest | Medicine, Research and Experimental |
| 339 | Cancer Gene Ther | Medicine, Research and Experimental |
| 340 | Cell Host Microbe | Microbiology |
| 341 | Plos Pathog | Microbiology |
| 342 | J Infect Dis | Microbiology |
| 343 | Cell Microbiol | Microbiology |
| 344 | Environ Microbiol | Microbiology |
| 345 | Mol Microbiol | Microbiology |
| 346 | Antimicrob Agents Ch | Microbiology |
| 347 | J Clin Microbiol | Microbiology |
| 348 | Int J Antimicrob Ag | Microbiology |
| 349 | Appl Environ Microb | Microbiology |
| 350 | J Bacteriol | Microbiology |
| 351 | Comp Immunol Microb | Microbiology |
| 352 | FEMS Microbiol Ecol | Microbiology |
| 353 | Eukaryot Cell | Microbiology |
| 354 | Protist | Microbiology |
| 355 | J Pineal Res | Physiology |
| 356 | Chronobiol Int | Physiology |
| 357 | J Mammary Gland Biol | Physiology |
| 358 | J Physiology | Physiology |
| 359 | Am J Physiol-Endoc M | Physiology |
| 360 | J Gen Physiol | Physiology |
| 361 | J Appl Physiol | Physiology |
| 362 | J Cell Physiol | Physiology |
| 363 | Am J Physiol-Heart C | Physiology |
| 364 | Am J Physiol-Cell ph | Physiology |
| 365 | Cell Physiol Biochem | Physiology |
| 366 | Physiol Genomics | Physiology |
| 367 | Exp Physiol | Physiology |
| 368 | Pflug Arch Eur J Phy | Physiology |
| 369 | Am J Physiol-Reg I | Physiology |
| 370 | Nat Biotechnol | Biotechnology and Applied Microbiology |
| 371 | Stem Cells | Biotechnology and Applied Microbiology |
| 372 | Mol Ther | Biotechnology and Applied Microbiology |
| 373 | Genome Biol | Biotechnology and Applied Microbiology |
| 374 | Nano Medicine-UK | Biotechnology and Applied Microbiology |
| 375 | Metab Eng | Biotechnology and Applied Microbiology |
| 376 | Biosens Bioelectron | Biotechnology and Applied Microbiology |
| 377 | Plant Biotechnol J | Biotechnology and Applied Microbiology |
| 378 | Bioinformatics | Biotechnology and Applied Microbiology |
| 379 | Microb Cell Fact | Biotechnology and Applied Microbiology |
| 380 | Bioresource | Biotechnology and Applied Microbiology |
| 381 | BMC Genomics | Biotechnology and Applied Microbiology |
| 382 | Biotechnol Biofuels | Biotechnology and Applied Microbiology |
| 383 | Mol Plant Microbe In | Biotechnology and Applied Microbiology |
| 384 | Pharmacogen Gen | Biotechnology and Applied Microbiology |
| 385 | J Nucl Med | Radiology, Nuclear Medicine and Medical Imaging |
| 386 | Radiology | Radiology, Nuclear Medicine and Medical Imaging |
| 387 | Neuroimage | Radiology, Nuclear Medicine and Medical Imaging |
| 388 | JACC Cardiovasc Imag | Radiology, Nuclear Medicine and Medical Imaging |
| 389 | Hum Brain Mapp | Radiology, Nuclear Medicine and Medical Imaging |
| 390 | Eur J Nucl Med Mol I | Radiology, Nuclear Medicine and Medical Imaging |
| 391 | Circ Cardiovasc | Radiology, Nuclear Medicine and Medical Imaging |
| 392 | Invest Radiol | Radiology, Nuclear Medicine and Medical Imaging |
| 393 | Int J Radiot Oncol | Radiology, Nuclear Medicine and Medical Imaging |
| 394 | Med Image Anal | Radiology, Nuclear Medicine and Medical Imaging |
| 395 | Radiother Oncol | Radiology, Nuclear Medicine and Medical Imaging |
| 396 | J Cardiovasc Magn R | Radiology, Nuclear Medicine and Medical Imaging |
| 397 | Contrast Medi Mol I | Radiology, Nuclear Medicine and Medical Imaging |
| 398 | Clin Nucl Med | Radiology, Nuclear Medicine and Medical Imaging |
| 399 | IEEE T Med Imaging | Radiology, Nuclear Medicine and Medical Imaging |
