# Supplementary material for: Misconduct Policies in High-Impact Biomedical Journals
Source: PLoS One. 2012 Dec 19;7(12):e51928. doi: 10.1371/journal.pone.0051928 (PMC3526485; doi:10.1371/journal.pone.0051928)
Supplement: Appendix S1 — Data Abstraction Form. (DOCX) [file pone.0051928.s002.docx]

**Appendix S1**. Data Abstraction Form

JOURNAL TITLE

DATE ACCESSED

IMPACT FACTOR (2010)

MEDICAL CATEGORY

EDITORIAL OFFICE SITE

PUBLISHER

ENDORSEMENT OF POLICY-PRODUCING BODY DEFINTIONS AND GUIDELINES

ICMJE COPE ORI WAME CSE OTHER NO

SOURCE OF MISCONDUCT POLICIES

JOURNAL PUBLISHER OTHER

DOES THE JOURNAL

MENTION THE TERM MISCONDUCT?

YES NO

HAVE A DEFINITION OF MISCONDUCT?

YES NO

FALSIFICATION FABRICATION PLAGIARISM DUPLICATION

DOES THE JOURNAL HAVE ANY POLICY REGARDING IMAGE INTEGRITY?

YES NO

DOES THE JOURNAL USE PLAGIARISM-CHECKING SERVICE?

YES NO

DOES THE JOURNAL HAVE PROCEDURES FOR RESPONDING?

YES NO

RETRACTION EXPRESSION OF CONCERN OTHER
